# Automatic bad-pixel mask maker for X-ray pixel detectors with application to serial crystallography

**DOI:** 10.1107/S1600576722009815

**Published:** 2022-11-21

**Authors:** Alireza Sadri, Marjan Hadian-Jazi, Oleksandr Yefanov, Marina Galchenkova, Henry Kirkwood, Grant Mills, Marcin Sikorski, Romain Letrun, Raphael de Wijn, Mohammad Vakili, Dominik Oberthuer, Dana Komadina, Wolfgang Brehm, Adrian P. Mancuso, Jerome Carnis, Luca Gelisio, Henry N. Chapman

**Affiliations:** a Center for Free-Electron Laser Science CFEL, Deutsches Elektronen-Synchrotron DESY, Notkestraße 85, 22607 Hamburg, Germany; b European XFEL GmbH, Holzkoppel 4, 22869, Schenefeld, Germany; c ARC Centre of Excellence in Advanced Molecular Imaging, La Trobe Institute for Molecular Sciences, La Trobe University, Melbourne, Australia; d Australian Nuclear Science and Technology Organisation (ANSTO), Australia; e Department of Chemistry and Physics, La Trobe Institute for Molecular Science, La Trobe University, Melbourne, Victoria, Australia; f Department of Physics, Universität Hamburg, Luruper Chaussee 149, 22761 Hamburg, Germany; g The Hamburg Centre for Ultrafast Imaging, Luruper Chaussee 149, 22761 Hamburg, Germany; SLAC National Accelerator Laboratory, Menlo Park, USA

**Keywords:** bad-pixel masks, robust mask maker, machine learning, robust statistics, serial crystallography

## Abstract

X-ray crystallography has witnessed a massive development over the past decade, driven by large increases in the intensity and brightness of X-ray sources and enabled by employing high-frame-rate X-ray detectors. The analysis of large data sets is done via automatic algorithms that are vulnerable to imperfections in the detector and noise inherent with the detection process. By improving the model of the behaviour of the detector, data can be analysed more reliably and data storage costs can be significantly reduced. One major requirement is a software mask that identifies defective pixels in diffraction frames. This paper introduces a methodology and program based upon concepts of machine learning, called robust mask maker (RMM), for the generation of bad-pixel masks for large-area X-ray pixel detectors based on modern robust statistics. It is proposed to discriminate normally behaving pixels from abnormal pixels by analysing routine measurements made with and without X-ray illumination. Analysis software typically uses a Bragg peak finder to detect Bragg peaks and an indexing method to detect crystal lattices among those peaks. Without proper masking of the bad pixels, peak finding methods often confuse the abnormal values of bad pixels in a pattern with true Bragg peaks and flag such patterns as useful regardless, leading to storage of enormous uninformative data sets. Also, it is computationally very expensive for indexing methods to search for crystal lattices among false peaks and the solution may be biased. This paper shows how RMM vastly improves peak finders and prevents them from labelling bad pixels as Bragg peaks, by demonstrating its effectiveness on several serial crystallography data sets.

## Abstract 2

Attention is focused on perhaps the biggest bottleneck in data analysis for serial crystallography at X-ray free-electron lasers, which has not received serious enough examination to date. An effective and reliable way is presented to identify anomalies in detectors, using machine learning and recently developed mathematical methods in the field referred to as ‘robust statistics’.

## 1. Introduction

X-ray crystallography using free-electron lasers (FELs) and synchrotron radiation has witnessed great progress over the past decade, partly due to the development of detectors that support increased frame rates and a higher number of pixels. Modern integrating X-ray detectors such as CSPAD (Hart *et al.*, 2012 ▸), AGIPD (Allahgholi *et al.*, 2015 ▸) and JUNGFRAU (Leonarski *et al.*, 2020 ▸), and counting detectors such as the PILATUS (Bech *et al.*, 2008 ▸) and EIGER (Dinapoli *et al.*, 2011 ▸), can record hundreds of megapixel diffraction patterns per second. Techniques like serial crystallography (our application example here; Chapman *et al.*, 2011 ▸) can generate petabytes of data per experiment, challenging data storage and analysis facilities technically and financially.

These X-ray detectors are not flawless and not all of the recorded data are useful. Often there are pixels in a detector that are damaged for various reasons (called bad pixels), corrupting parts of the collected data set. False signals generated by bad pixels can trigger monitoring systems to store uninformative volumes of data and bias analysis algorithms, producing low-quality results. Our aim is to develop a reliable and accurate method to detect the damaged or misbehaving pixels of an X-ray detector in order to (i) reduce the size of the collected data set to only the informative parts and (ii) improve the accuracy of the measurement so as to give better outcomes such as an improved resolution of the refined electron density.

**Figure 1:**
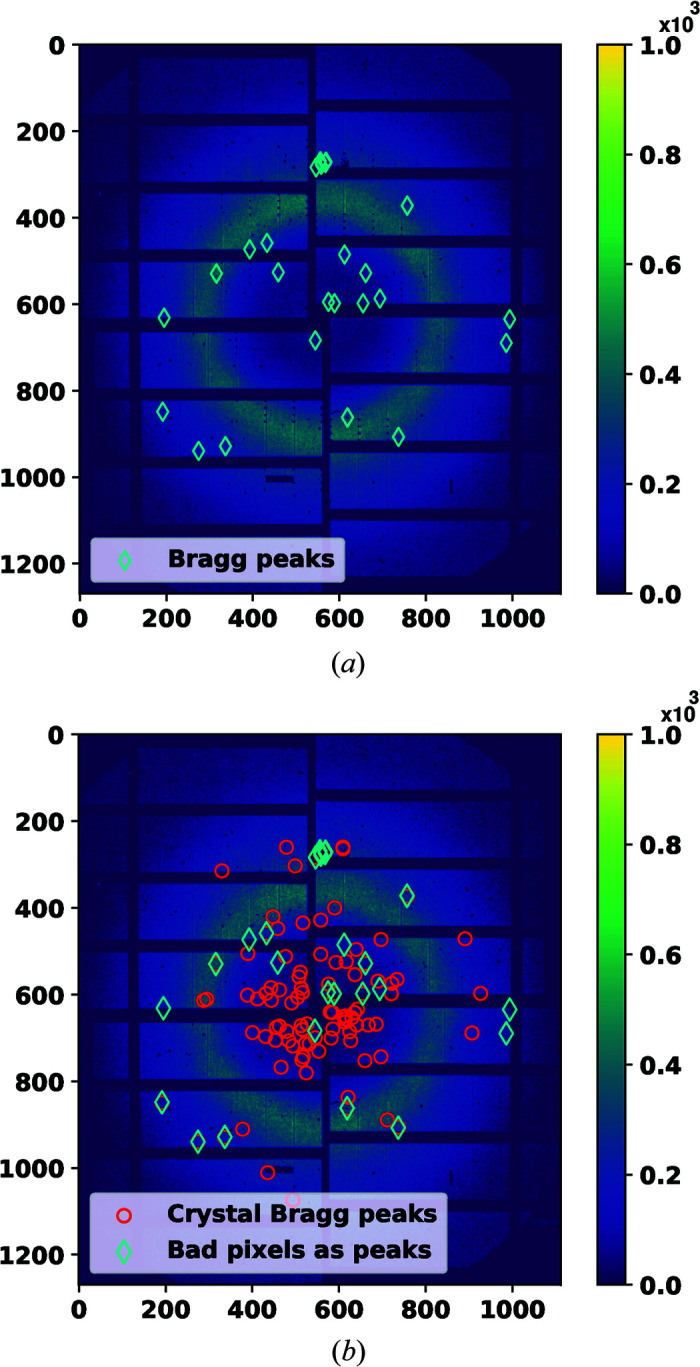
(*a*) A diffraction pattern of a liquid jet without any crystal. The peak finder software has identified bad pixels as Bragg peaks, marked by cyan diamonds. (*b*) A diffraction pattern of a crystal in the liquid where the same bad pixels as in panel (*a*) are still identified by the peak finding software as Bragg peaks. Those peaks also present in (*a*) are shown in cyan and the different peaks (which could be Bragg peaks belonging to one or more lattices) are shown by red circles.

An important first step in the analysis of diffraction patterns from crystallography measurements is the detection of Bragg peaks – which may be as small as one pixel – in the presence of background noise. This step is called peak finding. A monitoring computer program usually counts the number of detected Bragg peaks in each diffraction pattern and, if it is above a certain threshold, stores the pattern. Regardless of the improvements in peak finding methods (Barty *et al.*, 2014 ▸; Hadian-Jazi *et al.*, 2017 ▸, 2021 ▸), false signals from bad pixels (*e.g.* very bright spots or strong noise in the background) can still trigger peak finding methods to report incorrect Bragg peaks. To clarify this, an example of two different diffraction patterns from a serial crystallography data set is shown in Fig. 1 ▸. In this data set, the sample was a crystal of the lysozyme protein and data were collected using the AGIPD-1M detector. The data set is discussed in more detail in Section 4.1. A state-of-the-art Bragg peak finder called RPF (Hadian-Jazi *et al.*, 2021 ▸) was used to detect Bragg peaks in these diffraction patterns. Fig. 1 ▸(*a*) shows a diffraction pattern recorded when no crystal was present, yet the peak finder has identified a number of bad pixels as Bragg peaks, marked by cyan diamonds. Fig. 1 ▸(*b*) presents a diffraction pattern that includes Bragg peaks of a crystal hit by the X-ray beam. The set of peaks found in this figure includes the same bad pixels detected as peaks in Fig. 1 ▸(*a*). These have been manually identified on the basis that their locations are close to those found in the no-crystal pattern, and are marked again here as cyan diamonds. Bragg peaks that are not common to both patterns and which probably belong to one or more crystal lattices are marked by red circles. The peaks generated by bad pixels can be seen in most frames of the entire data set. As a result of including bad pixels in the list of Bragg peaks, the frame of Fig. 1 ▸(*a*) and others like it are stored and passed on to the indexing software, which then has to search for a non-existing crystal lattice in the pattern. This is time consuming, especially in an online analysis pipeline. Moreover, the presence of the spurious peaks in Fig. 1 ▸(*b*) prevents the indexing software from detecting the crystal lattice in that example. Using a binary software mask (the output of our program), data from such pixels can be excluded from analysis. Consequently, the frame depicted in Fig. 1 ▸(*a*) can be discarded and one crystal lattice could be detected in the frame depicted in Fig. 1 ▸(*b*). We will show in Section 4 that masking bad pixels results in a reduction in data and an improvement in analysis accuracy.

To help with data reduction and to improve the performance of the analysis, the bad pixels must be discovered and labelled on a regular basis during data collection, since pixels might become damaged or change their behaviour over the course of the experiment. This discovery is presently accomplished by repeatedly tuning the parameters of the available computer programs to search for bad pixels. Such exhaustive tweaking of parameters can be challenging and time consuming. The method we present here can analyse data to detect bad pixels accurately and generate bad-pixel masks with little to no parameter tuning.

The success of mask-making methods depends on two main factors: one is the availability of data sets exhibiting abnormal behaviour of bad pixels and the second is the algorithmic approach used to detect bad pixels. In this paper we discuss two kinds of data sets that are easy to collect, namely dark and bright flat fields, and a mathematical approach to analyse them. Recent improvements in statistical methods developed in the field of machine learning have been shown to be reliable for data analysis (Rousseeuw & Leroy, 2003 ▸; Huber & Ronchetti, 2011 ▸). Using these ‘robust statistics’ methods, it is possible to compare the behaviours of pixels with each other and divide them into two groups: those behaving normally and those that do not.

The structure of the paper is as follows. In Section 2, the abnormal behaviours of bad pixels relative to other pixels are discussed, and we show how to use these differences to detect them. The mathematical approach and the software for the detection of bad pixels are introduced in Section 3. In Section 4, our method is benchmarked against standard methods for three crystallography data sets.

## 2. Background

### 2.1. Bad pixels

Two types of detector are often used in X-ray crystallography experiments. The first type includes photon integrating detectors where the charge induced by the photon in the semiconductor of each pixel is accumulated (using an integrating circuit) and sampled regularly, and then the accumulator is discharged after sampling. The second type includes photon counting detectors where, if the accumulated charge passes a pre-set threshold, a counter is increased and the accumulating circuit is discharged to prepare for the next incoming photon. Both types have high numbers of pixel sensors and electronic elements to operate.

The photon integrating detectors provide the raw data of voltage values of each pixel sensor, even if these values do not reach the equivalent of a photon. We will discuss later that data collected in the dark field can be used effectively to calculate pixel offsets and model normal pixels. In photon counting detectors, the output values are non-negative natural numbers and a pixel value might not count up as a result of noise in the dark. As such, normal pixels usually show zero photon count most of the time in the dark field, which is not useful for calculating the variance of the data. Some abnormal pixels will still produce values that are too high compared with those of other pixels. In this paper our focus is on the integrating detectors such as AGIPD. However, we will show that bad pixels in the photon counting detectors are still detectable using our approach by assuming a reasonable minimum standard deviation for normal pixel values, set to 1/6.

The behaviour of pixels can change for a variety of reasons, including damage from high beam intensities, age or fabrication flaws. Each sensor pixel in an integrating or counting pixel array X-ray detector is composed of many electronic elements. During operation, some of these electronic elements can break down, making the readout value for some pixels behave abnormally. The data from such anomalous pixels must be masked. One way to make a suitable mask is to manually draw the regions to exclude. However, the number of defective elements in a detector can be very high, making this approach impractical. For example, AGIPD, used at the European XFEL, has 1024 × 1024 X-ray sensor pixels, each with 352 analogue memory cells to operate in burst mode. The CSPAD at LCLS has 2.4 megapixels. Counting detectors made by Dectris and used on the majority of crystallography beamlines, such as the PILATUS or EIGER, may have up to 16 megapixels. Moreover, some of these detectors support gain switching to provide increased dynamic range. For example, AGIPD supports three gain stages and provides the relevant data in the output. Switching the gain changes the analogue circuitry of the system, increasing the number of elements that can potentially fail.

One set of bad pixels are those at the edges of sub-modules of the detector array called ASICs. These pixels usually vary in shape, size and sensitivity. The edge pixels are made to be insensitive to X-rays and are often used as in-built calibration elements. In our approach, such pixels are masked without analysis (Allahgholi *et al.*, 2015 ▸).

A bad pixel can appear in many frames in sequence as a bright spot compared with adjacent pixels. To find the bad pixels, the detector can be illuminated by a uniform X-ray beam, such as that generated by the X-ray fluorescence of a foil, where all pixels receive similar X-ray counts. The data collected over a series of frames can be used for the detection of pixels with abnormal behaviour. When the detector is not illuminated by any X-ray beam (to give what are termed dark frames by closing the detector’s shutter), bad pixels might present very high or very low readings.

### 2.2. Bad-pixel masking

There is software available to generate bad-pixel masks for megapixel X-ray detectors, including the mask maker in *Cheetah* (Barty *et al.*, 2014 ▸) and the *CSPADMaskMaker* (LCLS, https://confluence.slac.stanford.edu/display/PSDM/Mask+Editor). While the *CSPADMaskMaker* provides a GUI for manual interaction, both programs also offer statistical methods to find bad pixels. The methods and algorithms in these programs use non-robust statistics which can lead to masking good pixels and missing bad pixels. In order to prevent that, the programs rely on the tuning of input parameters, which in turn makes the process difficult for users and may give sub-optimal results, especially when used for automated data reduction. In this paper, we compare the results of our method, called robust mask maker (RMM), with those obtained using the mask provided in the published data sets, made by the programs mentioned above.

### 2.3. Robust model fitting and outlier detection

In the approach presented here, detector pixels are divided into two groups (normal and abnormal pixels) by fitting a simple geometric model with Gaussian noise to a series of detector frames. What we refer to here as a detector frame, or image, is an array of pixel values with the indices of each value given by the location of the pixel in the detector. The fitting, however, needs to be done in the presence of bad pixels which are yet to be identified. Robust statistics methods can deal with geometric fitting in such situations where the data can be divided into inliers (here normally behaving pixels) and outliers (here bad pixels), the model being fitted to inliers only. The challenge is to avoid fitting the model to outliers, which is what non-robust methods cannot do since they use the entire data set regardless of the presence of bad pixels. Here we use two basic methods to calculate robust statistics of the data. The first consists of robustly fitting geometric models to the data and the second is to obtain a noise scale for normal pixels (inliers) that allows separation of bad pixels (outliers). The underlying mathematical behaviour of these methods is reviewed in detail in Appendix *A* [App appa].

Since geometric model fitting using a Gaussian probability density function has many potential applications in the analysis of FEL imaging data, a software library including the methods mentioned above has been made available, named *RobustGaussianFittingLibrary* (Sadri, 2020 ▸). Using this library, the X-ray data sets were analysed and bad pixels were identified as explained in Section 3.

## 3. Method

Sequences of detector frames collected under controlled X-ray illumination can be used to generate a bad-pixel mask. To do this, frames are acquired under dark and bright flat fields. By flat fields, we mean that the majority of pixels in subsets of the data set are expected to have similar statistical characteristics which are called features. In a flat field, a subset of good pixels contains those that have similar feature values whose variance can be modelled with reasonable accuracy with a Gaussian or Poisson probability density function (more generally, approximately bell shaped). This expectation allows us to use robust statistics.

In this section we first discuss the operator applied to any given vector of feature values for a subset of pixels to partition them into good and bad pixels. This is done via a function that takes an arbitrary input vector and calculates the robust statistics of the input vector. The output of the function is a set of labels that can place the pixels associated with the input into two groups.

Afterwards, the paper continues with introducing how different features are extracted and provided as input to the robust operator to make different masks. Note that the number of features that can be extracted from the data is unlimited, although in this paper we refer to just a few that have yielded good results in our tests.

The section finishes by introducing the RMM procedure that collects and combines the set of masks from different features to create the final bad-pixel mask. Note that the mask will be provided for each pixel, for each panel, for each memory cell and for each gain stage individually, depending on the detector.

### 3.1. Outlier detection function

The core message of robust statistics is that it is not the bad pixels that are being modelled. Rather, the statistics describing groups in the data are based on modelling the normal behaviour of good pixels. In this part, a function is introduced that uses robust statistics to fit simple geometric models to normally behaving pixels with a Gaussian noise model. The input of this function is a vector of feature values for each pixel. As will be seen later, these feature values may be the intensity values themselves, or a statistic based on groups of related pixels (in one or many frames). The output of the function is a set of labels, good (1) for inliers of the Gaussian or bad (0) for outliers, corresponding to each element of the input.

Given an input vector of feature values for a subset of *N* pixels, *x* = {*x*(*i*)}, *i* = {1,…, *N*}, robust statistics can be used to find the robust average 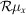 and robust scale of the Gaussian 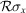 ( 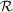 refers to the robustness of the statistics). Given the Gaussian model robustly fitted to the subset, a statistical separability called SNR is calculated for each pixel of the subset, according to the definition 

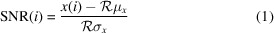

for the *i*th pixel in the input vector. This value reduces the problem of bad-pixel detection into a simple statistical question: how far from its mean can samples of a normal probability density function (normalized by its standard deviation) be considered as inliers? This question is parametrized by λ. In other words, *x*(*i*) is an inlier if |SNR(*i*)| < λ. This can be used to define an outlier (a bad pixel) with the feature value of *x*_O_ that is separable from the inliers if 

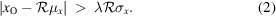

Note that we have no intention of modelling the behaviour of bad pixels (hence the name ‘outliers’). For example, the value of a bad pixel over time (among many consecutive frames) may be constant and show zero variance, or it may show an extreme bi-modal behaviour switching between very high and very low values. We propose to mask pixels whose behaviour does not fit into the normal behaviour of the majority of pixels in the subset. The subset *x* mentioned above can be as large as all pixels of all frames or as small as pixels within a local region in one frame, depending on how we expect the pixels to behave normally.

The default value of the input parameter λ is usually unknown in the statistics literature. But it is clear that, if it is set to lower values, fewer inliers will be included in the estimation of model parameters, which can lead to inaccuracy. In Appendix *B* [App appb] we will show, by example, how changing λ affects the overall results at the end of the analysis pipeline and suggest a default value of λ = 8.0.

### 3.2. Detector in the dark

One of the data sets that is usually readily available is that of data collected in the absence of X-ray illumination. When the shutter is closed, the pixels will show their offsets from zero. Different features can be extracted for different detectors out of pixel values in such a data set. Here we list some that can be extracted using the detectors we studied for this research.

#### 3.2.1. Feature 1: mean intensity of frames

The dark-field data set is usually composed of a large set of frames which will also be used for calibrations. Frames for each gain stage are required for multi-gain integrating detectors, and for each memory cell for detectors with analogue memory cells. The dark-field data set is collected to provide offsets for all analogue readouts of an integrating detector. This data set is mainly used for the calibration of detectors.

Here we define a feature that is used to flag frames that should be excluded from further analyses of bad pixels. It is a scalar value for each frame, representing the mean intensity of each frame for each gain stage and memory cell. It is calculated from the robust average of all pixels in a frame, giving the set *x*_F1_ = {*x*_F1,*t*_} = 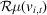 for frames *t* = 1,…, *T*, where *v*_*i*,*t*_ is the intensity value of pixel *i* in frame *t*. Then, from this set *x*_F1_ we use the robust estimator [equation (2)] to detect and label outlying frames. The frames labelled as outliers are discarded from the dark-field data set in the remainder of the analysis and generation of the bad-pixel mask.

After outlying frames of the data set have been rejected, the remaining reliable frames are analysed at the level of individual pixels to extract particular features to make the bad-pixel mask, as described in the following sections.

#### 3.2.2. Feature 2: pixel offset values

The read-out values of pixels in an integrating detector like the AGIPD or JUNGFRAU are proportional to the total energy of the photons deposited into the sensor of the pixel. The constant of proportionality is referred to as the gain, and pixels usually exhibit an offset value when there is no photon exposure. Although this offset is often calibrated, it may drift and must be accounted for. However, it often happens that a broken pixel shows an abnormal offset value. Feature 2 is the offset value in the dark value recorded by each pixel for each gain stage, memory cell, module and ASIC of an integrating detector. The offset value for each pixel is calculated by the non-robust average over *T* frames and the elements of the set of offset values of all pixels are used as the feature set given to the outlier detection function, *i.e.* *x*_F2_ = {*x*_F2_(*i*)} = 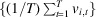 , *i* = (1,…, *N*). We then apply robust Gaussian fitting to find the robust average and standard deviation of this set. Then, by comparing the elements of the set with the robust average and standard deviation, we find SNR_F2_(*i*) for each pixel using the procedure described above with a value of λ = 8. Pixels with {SNR_F2_(*i*) > λ} are labelled bad pixels.

**Figure 2:**
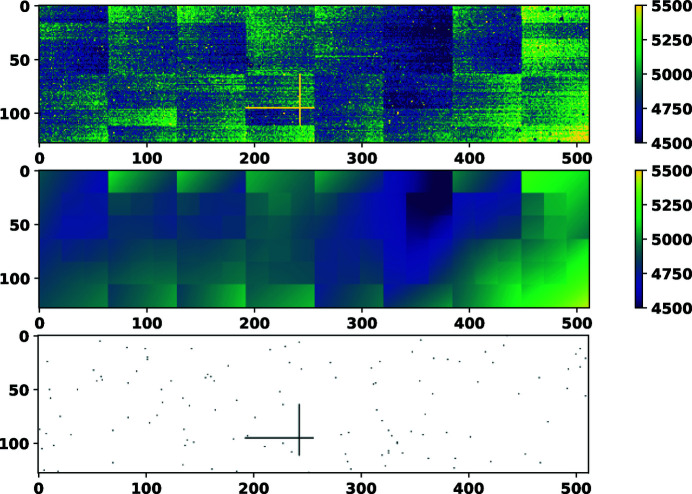
(Top) The temporal average values of a module of AGIPD-1M read in the dark, which is Feature 2 or the set *x*_F2_. (Middle) The robust model values at the location of each pixel, estimated to declare normal behaviour (estimated over pixels within 64 × 64 windows). (Bottom) Detected bad pixels, shown in black.

An example of the above procedure for the AGIPD-1M detector, module 4, memory cell 1 in the high-gain stage is shown in Fig. 2 ▸. In this image the values for Feature 2 [that is, *x*_F2_(*i*)] as given to the robust estimator function are shown in the top panel. The robust estimator function models the pixel values, and the values of the model at the locations of the pixels are shown in the middle panel. It is seen that this map is free of noise and outliers since it is not affected by the presence of outliers (which shows the robustness of the statistics). It shows the average offsets of normally operating pixels which are seen to change smoothly from pixel to pixel within certain blocks of differing sizes. These blocks correspond to the ASICs of the detector. The set of abnormal pixels, as determined using equation (2), are marked in black in the bottom panel.

#### 3.2.3. Feature 3: pixel variations

The variance of noise in the pixel values under dark conditions is usually different for each pixel. Too much (abnormal) variation of the dark values is usually problematic and may obscure the measured signal. The variation of the dark values is calculated as the non-robust standard deviation over all frames for each pixel, and the set of these values is then given to the outlier detection function to find SNR_F3_(*i*) for each pixel *i*, *i.e.* *x*_F3_ = {*x*_F3_(*i*)} = 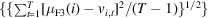 , where the mean μ_F3_(*i*) = 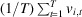 is the non-robust average over all *T* frames for pixel *i*. The set of outliers for which {|SNR_F3_(*i*)| > λ} are considered to behave abnormally and are masked as bad pixels according to this feature.

#### 3.2.4. Features 4 and 5: gain stage indicator values

To increase their dynamic range, gain switching detectors such as AGIPD can switch the gain of the amplification of the analogue signal. The AGIPD, for example, provides an output that is used to indicate which gain stage the pixel switched into, digitized as a 14-bit number. In order to obtain these values for different gain stages, data must be collected while the detector is operating in each of those stages. Except when switching gains, these values ideally should remain constant over time, exhibit low noise and be different for each gain stage. For a group of bad pixels, the value of the output does not always make a large enough change to distinguish the gain stage that the pixel used. Such abnormally behaving pixels do not allow this discrimination and are detected using the following two features.

(i) *Feature 4: between gain stages.* The current gain-switching detectors used at XFEL facilities may display misleading gain stage values for bad pixels. The non-robust average 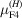 = 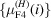 = 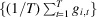 (where *g*_*i*,*t*_ is the gain stage indicator value of the *i*th pixel of frame *t*) and the standard deviation 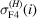 = 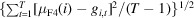 are used to model the value of the indicator for a pixel in the dark over time (for each individual memory cell) for the gain stage *H* (*e.g. H* can represent high-, medium- and low-gain modes for AGIPD). For normal functioning pixels, it is expected that the averages of the values of one stage are independent of those of other stages. Without using further statistics, the pixels that do not present distinguishable values for all of their gain stages (for each and all *H*) are simply masked, *i.e.* the set for which 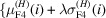 > 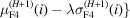 are masked according to Feature 4.

(ii) *Feature 5: for each gain stage.* Another feature used to identify bad pixels is the average of the gain indicator values in the dark, 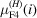 . This value for bad pixels is abnormally different from the majority of pixels. We propose comparing pixels with each other based on the temporal average of the gain stage indicator values for each pixel in each stage (for each memory cell). To do that, the outlier detection function is called with its input set as *x*_F5_ = {*x*_F5_(*i*)} = 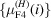 . A Gaussian is fitted to these values robustly and the robust average 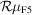 and standard deviation 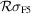 are found to define SNR_F5_(*i*) according to equation (1). The set of outliers of this Gaussian, {|SNR_F5_(*i*)| > λ}, is considered to contain pixels that are behaving abnormally and will be masked.

**Figure 3:**
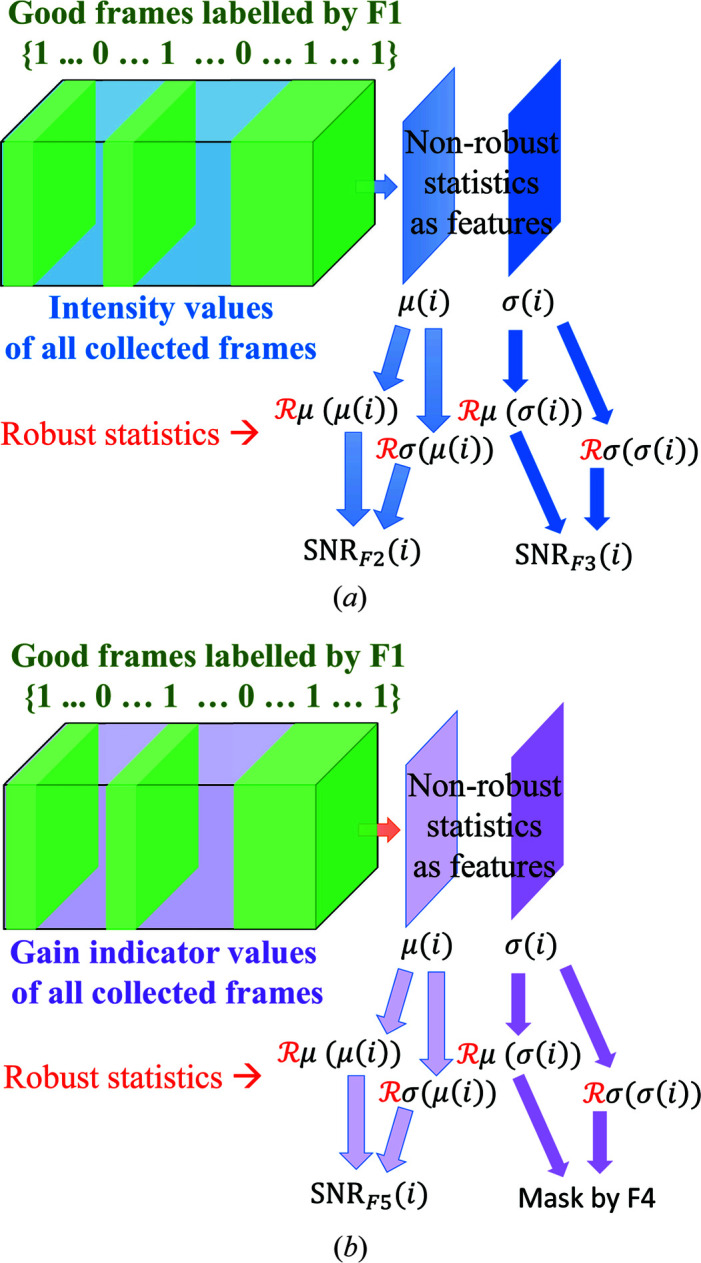
The procedure for calculating different SNR values for every pixel using data from the dark field for a multi-stage detector, such as AGIPD. (*a*) The analysis of the data set of intensity values. (*b*) The analysis for gain stage indicator values if available. In both, analysis starts by reducing the size of the input data set to frames that present normal overall intensities according to Feature 1 (F1). The non-robust statistics are used as features for each pixel and pixels are compared with each other afterwards. Each mask is generated by analysing features F1 to F5 as explained in Section 3.2 and combined as explained in Section 4.4.

To summarize the generation of a bad-pixel mask using the dark-field data of an integrating detector, a flow chart is presented in Fig. 3 ▸. The analogue pixel values and gain indicator values obtained in the dark field are shown as two tensors of data. Frames with normal overall intensities (detected using Feature 1, F1) are shown in green and are used for further feature extractions and calculation of the bad-pixel mask. First, the non-robust temporal averages and standard deviations are obtained. Then a geometric model is fitted robustly to these quantities and SNR values for every pixel are calculated, associated with each feature. A pixel is masked if the absolute value of *any* of these SNR values is above a given global threshold λ.

### 3.3. Detector under light

#### 3.3.1. Feature 6: average SNR of pixels over time

It often happens that pixels that show no abnormal behaviour in the dark still behave abnormally when illuminated by X-rays. The intensity values of these pixels can be too high or too low most of the time, regardless of the overall intensity of the beam. In the presence of uniform illumination, even though all pixels might present values that occasionally rise above an acceptable SNR(λ), some might do so too often. If not masked, such pixels may be flagged as pixels with sensitive information and become an obstacle for the rest of the analysis pipeline and data reduction. As such, a large data set of frames for each gain stage, for each memory cell, for each detector module and for each pixel (depending on the detector) needs to be gathered with incident illumination that is approximately uniform across the detector. The RMM program presented in this paper does not need the X-ray illumination to be truly flat, as long as it does not cause sudden bright spots in one or a few pixels. Also, since a robust estimator is used at this stage, there is no need for a very large data set to be collected (arguably, even 200 frames are sufficient for each memory cell, for each module and for each gain stage; Hoseinnezhad *et al.*, 2006 ▸).

RMM calculates the statistical separability of every pixel from its background for every frame collected under X-ray illumination. For a frame *t* and a given window *W*_*i*,*t*_ of pixels located around a pixel in that frame, the set of pixel values {*v*_*j*,*t*_, *j* ∈ *W*_*i*,*t*_} is used for robustly finding the parameters of the Gaussian model, 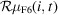 and 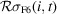 . While it is suggested that it should be as large as possible, the size of the local area *W*_*i*,*t*_ can include as few as 200 pixels (windows of size 15 × 15) without losing accuracy, as recommended by Hoseinnezhad *et al.* (2006 ▸). To find the robust average 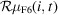 , a plane is first fitted to the background values *v*_*j*,*t*_ of the local region to give μ_F6_(*i*, *t*) as the projection of the *i*th pixel onto that plane. SNR_F6_(*i*, *t*) is then found and its non-robust average over all frames is chosen as the input feature of the outlier detection function to result in calculation of SNR_F6_(*i*). If the value of a pixel is excessive too often, so as to produce (abnormally) high SNR values, the pixel is masked. A similar procedure is followed for pixels showing abnormally low values.

The final overall mask is simply composed of pixels labelled bad following any of the above calculations. The software is set up accordingly, as explained in Section 4.4.

## 4. Results

We applied RMM to three crystallographic data sets to evaluate its performance. If not treated correctly, bad pixels that are too bright compared with their surroundings will be identified as Bragg peaks. Also, bad pixels with intensities that are too low can bias the estimated model parameters for Bragg peaks, depending on the robustness of the Bragg peak finder (Hadian-Jazi *et al.*, 2021 ▸). We compared the results of data analysis using the published masks available in the data sets with results using masks generated by RMM. The influence of using the two bad-pixel masks is highlighted by comparing the crystallographic self-consistency parameters of the data sets after indexing and merging of patterns. In the following, the mask was changed in the analysis pipeline while all other methods or parameters were fixed. The figures of merit used to compare the performance of the bad-pixel masks are CC*, *R*_split_, CC_1/2_ and SNR, which are commonly used as quality indicators in crystallographic data analysis pipelines (Karplus & Diederichs, 2015 ▸).

In the crystallographic data analysis pipeline, the peak-finding stage is the most vulnerable to bad pixels, and the choice of peak finding method can affect the sensitivity of the pipeline to bad pixels as well. The reason is that peak finding methods are typically used to analyse frames individually and no information is shared between frames. As such, a peak finding method has no prior knowledge that a pixel shows extreme values abnormally or too often. We used the two peak finding methods PF8 (Barty *et al.*, 2014 ▸) and RPF (Hadian-Jazi *et al.*, 2017 ▸) as they are based on two different approaches to peak finding. PF8 uses a non-robust yet iterative outlier filtering technique to detect Bragg peaks, and it is expected that bad pixels will bias the non-robust estimates of the background, preventing the method from detecting actual Bragg peaks. RPF uses robust statistics for modelling the background and detecting Bragg peaks. While its models are not biased by bad pixels, those bad pixels that show abnormally high values are likely to be picked up as Bragg peaks unless they are masked beforehand. For indexing and calculating the figures of merit, *CrystFEL* (Version 0.9.1; White *et al.*, 2012 ▸) was used. In the following sections the results of the tests on the analysis of three different crystallographic data sets are provided in terms of self-consistency values. It is also useful to study the effect of the mask on the hit rate (the number of hits out of all frames in the data set; hits are frames when the beam hits a crystal) and on the indexing rate (the number of patterns indexed by the crystallography suite out of all the hits).

### 4.1. AGIPD-1M, SPB/SFX, EuXFEL

**Figure 4:**
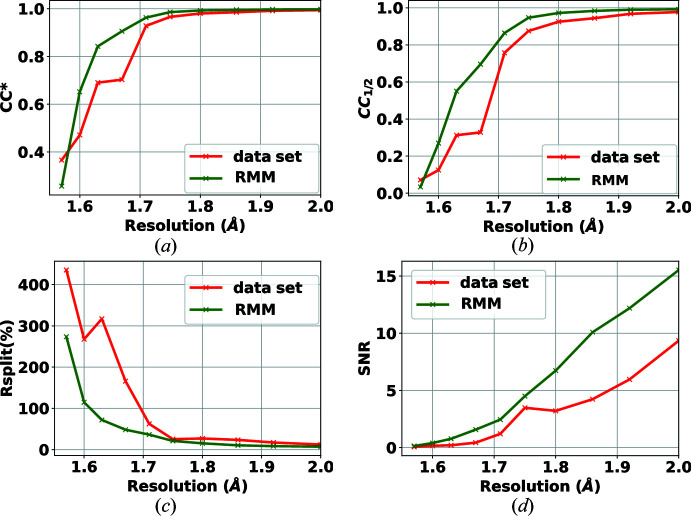
Effect of RMM on the self-consistency statistics (*a*) CC*, (*b*) CC_1/2_, (*c*) *R*_split_ (%) and (*d*) SNR, using the PF8 peak finder for the detector AGIPD 1-M. Green curves show the results when applying the proposed bad-pixel mask, while the red curves show the results when using the bad-pixel mask provided with the data set.

In this experiment, the target samples were crystals of lysozyme. The data set was collected in March 2020 ▸ as an experiment for commissioning the AGIPD detector at the EuXFEL on the SPB/SFX instrument (Mancuso *et al.*, 2019 ▸). X-ray pulses of 9.3 keV photon energy were delivered at a repetition rate of 1.1 MHz within bunch trains (352 pulses per bunch train as AGIPD has 352 analogue memory cells, trains delivered at 10 Hz). The AGIPD-1MP detector (Allahgholi *et al.*, 2015 ▸) was 129 mm away from the jet injecting the crystals into the X-ray beam. The sample was delivered using a 3D-printed gas dynamic virtual nozzle (Knoška *et al.*, 2020 ▸) in combination with injection infrastructure described by Vakili *et al.* (2022 ▸). The data set is composed of seven runs. Three runs include patterns collected in the dark, one for each of the gain stages of AGIPD. That is, for each gain stage and for each memory cell of AGIPD (352 cells), 512 frames were collected and reduced to 200 normally biased frames, as explained in Section 3.2.1. Another run contains bright near-flat field data which helps in detecting bad pixels that are not detected in the dark field. Three further runs include experimental crystallography data (collected while all memory cells and gain stages were active) with a total of 5.5 million diffraction patterns. The results for self-consistency statistics for peak finders PF8 and RPF are shown in Figs. 4 ▸ and 5 ▸, respectively. For both peak finders, all of the crystallographic parameters (CC*, *R*_split_, CC_1/2_ and SNR) are improved when the RMM bad-pixel mask is used. These results verify that the detector includes bad pixels that need to be masked and that the methodology introduced in this paper is reliable and effective. The advantage of using the proposed method to generate a bad-pixel mask is that the process is automatic and requires little to no parameter tuning.

One question that remains unanswered is the importance of the robustness of the statistics. A contribution of our work is to automate the process of bad-pixel mask generation. However, to reduce the sensitivity of the algorithm to input parameters and to increase the accuracy of the analysis, robustness of the algorithm is crucial. As such, we performed a test using only non-robust statistics, to produce what we refer to as a non-robust mask. Table 1 ▸ provides the hit and indexing rates of the analysis for the mask published with the data set, for our method without robustness and for our method with robustness (labelled RMM). As can be seen, the number of hits increases dramatically as a result of using a non-robust mask. These hits found using a non-robust mask are false positives. This result proves the effectiveness of the proposed method for the task of data reduction. The results in Figs. 4 ▸ and 5 ▸ show the improvement in accuracy in PF8 and RPF when the RMM bad-pixel mask is used. Our conclusion is that the hit rate is overestimated if no mask is applied or when using a mask generated based on non-robust statistics. In these figures we only report the metrics up to 2 Å, as the values of these curves do not change trend at lower resolutions.

### 4.2. CSPAD-2.5M, CXI, LCLS

**Figure 6:**
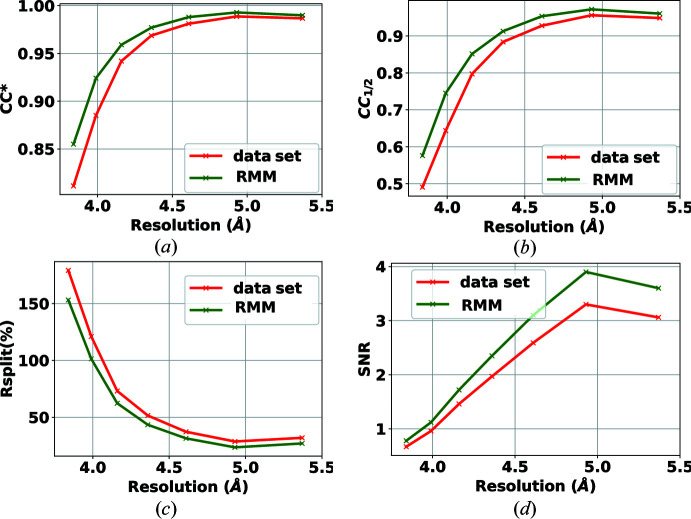
Effect of RMM on the self-consistency statistics (*a*) CC*, (*b*) CC_1/2_, (*c*) *R*_split_ (%) and (*d*) SNR, using the PF8 peak finder for the detector CSPAD 2.5-M. Green curves show the results when applying the proposed bad-pixel mask, while the red curves show the results when using the bad-pixel mask provided with the data set.

The second data set, containing diffraction from crystals of the rhodopsin–arrestin complex, was collected with a CSPAD detector on the CXI beamline at the Linac Coherent Light Source (LCLS) (Zhou *et al.*, 2016 ▸). The data set is publicly available in the Coherent X-ray Imaging Data Bank (Maia, 2012 ▸) (entry 32). The CSPAD detector has 32 modules of 185 × 388 pixels each (2.5 megapixels in total). The published data set includes a bad-pixel mask made by the software *CSPADMaskMaker*. Our proposed automated mask maker uses the dark runs and a run that contains no crystal frames to determine a bad-pixel mask. We applied our mask to the two million frames of the data set, and the results for the self-consistency statistics are shown in Figs. 6 ▸ and 7 ▸ using peak finders PF8 and RPF, respectively. The results have improved using the RMM mask for both peak finders. This shows that the proposed bad-pixel mask method can be used reliably for different detectors and experiments while improving the results of the data analysis.

In Table 2 ▸, the hit and indexing rates are provided. The use of the mask has an overall positive effect on the reduction of the hit rate (by reducing false positive hits) and an increase in the indexing rate (by finding true hits). This shows that applying the right mask will avoid overestimating the hit rate, and in this case increase the indexing rate. This is especially important for online/immediate analysis of the data. By considering the results in Figs. 6 ▸ and 7 ▸ one can also observe an improvement in accuracy.

### 4.3. PILATUS-6M, PETRA III, P11, DESY

**Figure 8:**
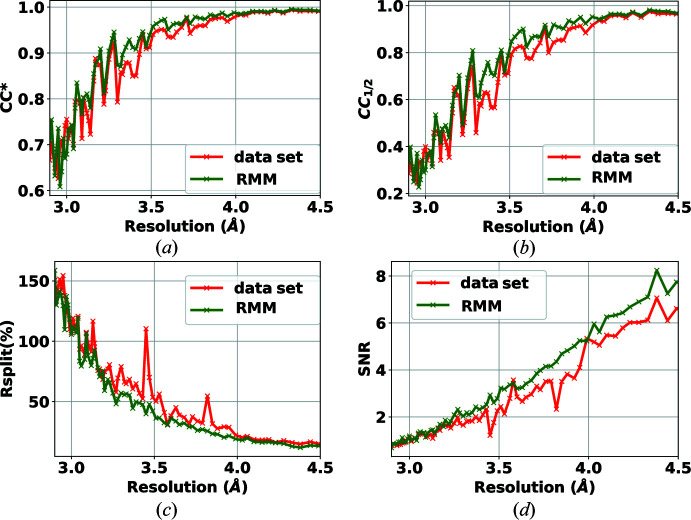
Effect of RMM on the self-consistency statistics (*a*) CC*, (*b*) CC_1/2_, (*c*) *R*_split_ (%) and (*d*) SNR, using the PF8 finder for the detector PILATUS 6-M. Green curves show the results when applying the proposed bad-pixel mask, while the red curves show the results when using the bad-pixel mask provided with the data set.

This experiment recorded diffraction patterns of a dioxygenase mixture using a PILATUS 6M detector. PILATUS is a photon counting detector, unlike the integrating detectors used in the experiments of Sections 4.1 and 4.2. The experiment was performed using 12 keV X-rays and the detector was positioned 250 mm downstream of the sample. The collected data were divided into many data sets where different experimental settings were tested, some of which are discussed in the relevant paper (Beyerlein *et al.*, 2017 ▸). A subset of this data set, chosen for our evaluation, is composed of hits that include very weak Bragg peaks. Peak finders often set a minimum threshold of the SNR of a peak. For this experiment, we set this to a low value of 5 to be sensitive to weaker peaks. In such a scenario, the presence of bad pixels can drastically reduce the performance of peak finders. We chose this subset from the entire data set as this would be a challenging data set to test our proposed methodology. The dark runs available in the data set were used to make the bad-pixel mask. The data set includes 453 231 frames, of which only a small portion are indexable. The self-consistency statistics are shown in Figs. 8 ▸ and 9 ▸, demonstrating that RMM has improved the analysis. The numbers of hits and indexed patterns are listed in Table 3 ▸. This challenging data set contains fewer frames than the last two. The use of the mask has a positive effect on the reduction in the hit rate and, even in this special case, results in an increase in the indexing rate and, critically, the number of indexed patterns.

### 4.4. Software

RMM is implemented in Python Version 3 and is publicly available for the generation of bad-pixel masks (Sadri, 2021 ▸). The software documentation provides guidance on how to add support for other X-ray detectors. The core robust statistics methods are implemented in a software package that we have called *RobustGaussianFittingLibrary*.

One of the key benefits of RMM is that each module of the detector can be analysed in parallel, making the algorithm scalable on cluster computers. The output file contains an 8-bit value for every pixel. A value of 0 indicates good pixels. Other values above 0 present a specific problem for each pixel according to the features introduced in Section 3.

## 5. Conclusions

We have introduced a methodology for generating bad-pixel masks for X-ray pixel detectors, called robust mask maker (RMM), that is based upon modern approaches of machine learning. When working with detectors with multiple gain stages and multiple memory cells, RMM requires two sets of data: one recorded in the dark (that is, without X-rays) and one recorded with a nearly uniform exposure (such as from X-ray fluorescence from a foil). These two data sets should pan each gain stage and memory cell. Different measurements are obtained from the dark data set, such as offset or variation in the dark. The average SNR of a pixel under X-ray exposure is also calculated as another feature for each pixel. Considering that these measurements are supposed to be almost the same for adjacent pixels, we use robust statistics to segment out pixels that behave normally and mask out the abnormal ones.

We have evaluated and compared the performance of the proposed method with existing methods using three crystallographic data sets, each collected with a different kind of detector. The data were passed through the analysis pipeline for crystallography to examine the effect of the analysis on the bad-pixel mask. When using an inaccurate mask, some bad pixels might be detected as Bragg peaks by the peak finding software. In such cases, serial crystallography analysis software tends to overestimate the hit rates, which in turn causes a low indexing rate. More importantly, the number of indexed patterns may only be a fraction of the possible indexable patterns. We found that the improved RMM bad-pixel mask often increased the total number of indexed patterns, even though fewer patterns were identified as hits. Moreover, by using an improved mask we have greatly speeded up the indexing since we are not feeding the analysis software un­informative patterns. RMM is relatively fast and scalable and shows a low sensitivity to its parameters (over a wide range of values), which makes it suitable for automatic processes and the task of online/offline data reduction.

## Figures and Tables

**Figure 5:**
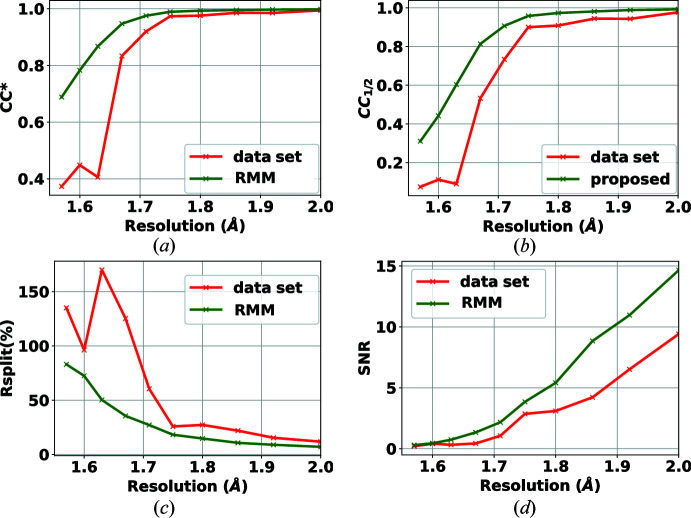
Effect of RMM on the self-consistency statistics (*a*) CC*, (*b*) CC_1/2_, (*c*) *R*_split_ (%) and (*d*) SNR, using the RPF peak finder for the detector AGIPD 1-M. Green curves show the results when applying the proposed bad-pixel mask, while the red curves show the results when using the bad-pixel mask provided with the data set.

**Figure 7:**
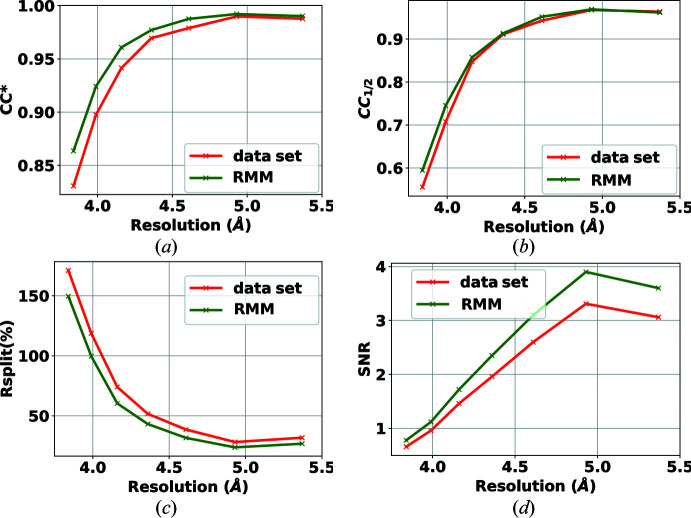
Effect of RMM on the self-consistency statistics (*a*) CC*, (*b*) CC_1/2_, (*c*) *R*_split_ (%) and (*d*) SNR, using the RPF peak finder for the detector CSPAD 2.5-M. Green curves show the results when applying the proposed bad-pixel mask, while the red curves show the results when using the bad-pixel mask provided with the data set.

**Figure 9:**
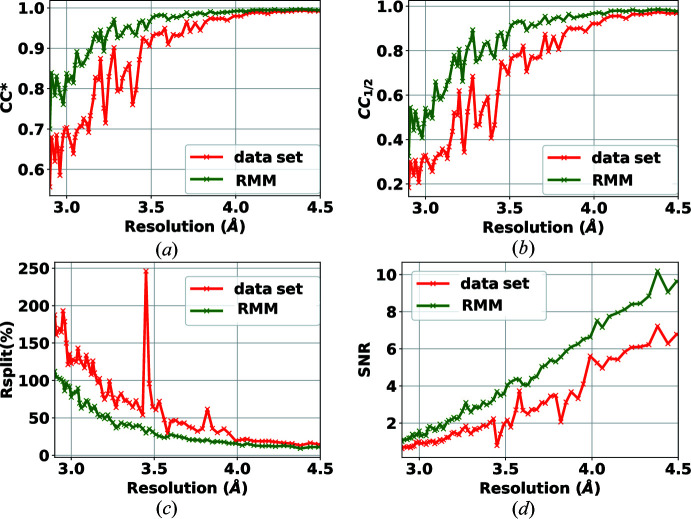
Effect of RMM on the self-consistency statistics (*a*) CC*, (*b*) CC_1/2_, (*c*) *R*_split_ (%) and (*d*) SNR, using the RPF peak finder for the detector PILATUS 6-M. Green curves show the results when applying the proposed bad-pixel mask, while the red curves show the results when using the bad-pixel mask provided with the data set.

**Figure 10:**
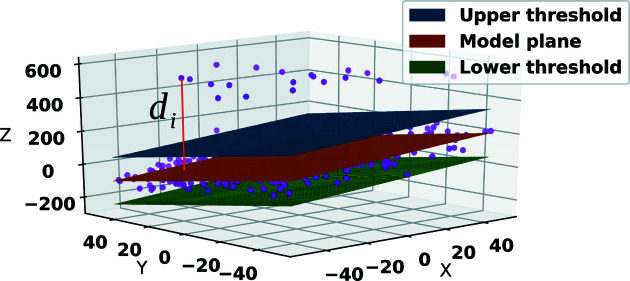
Robust fitting of a plane to a set of 3D data points (in purple). The outliers have no effect on the model of the plane (background) where the majority of the data behave normally. The algebraic error (shown in red for a data point *d_i_*) is the model fitting residual for any data point.

**Figure 11:**
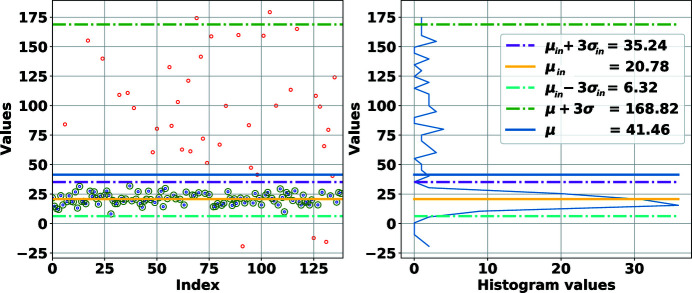
Robust fitting of a Gaussian to a set of 1D data points. The inliers are shown as small blue circles and outliers as small red circles. Detected inliers are marked with larger green circles. The blue curve presents the distribution of the data. The robust average, upper and lower thresholds using the fitting method are shown with yellow, magenta and cyan lines, respectively. The non-robust average and upper threshold are also shown. The lower non-robust threshold is too low and off the chart.

**Figure 12:**
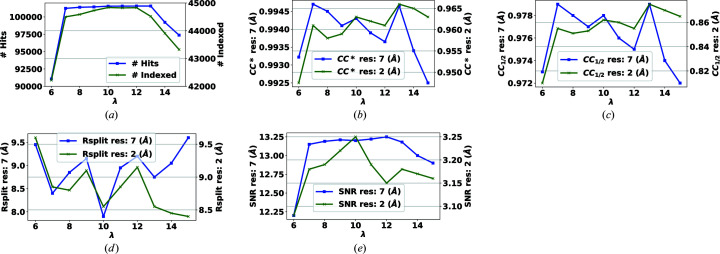
The effect of changing the threshold for abnormalities of pixels on the self-consistency statistics for a subset of the data set introduced in Section 4.1.

**Table 1:** An overview of numerical results related to the analysis in Section 4.1 (AGIPD detector) Total number of frames 5 764 736.

Procedure	No. of hits	Hit fraction (%)	No. of indexed frames	Indexing rate (%)
RPF data set mask	5 701 046	98.89	1 485 668	26.05
RPF with non-robust mask	4 032 906	69.95	1 391 527	34.50
RPF with RMM	2 527 171	36.89	1 285 649	70.65
PF8 data set mask	5 117 076	88.76	1 496 030	29.23
PF8 with RMM	3 422 532	59.37	1 257 048	51.33

**Table 2:** An overview of numerical results related to analysis in Section 4.2 (CSPAD) Total number of frames 3 135 659.

Procedure	No. of hits	Hit fraction (%)	No. of indexed frames	Indexing rate (%)
RPF with data set mask	410 445	13.08	31 783	7.74
RPF with RMM	58 670	1.87	54 345	92.62
PF8 with data set mask†	375 288	11.96	23 528	6.26
PF8 with RMM	37 466	1.19	36 369	97.07

† Reproduced using the output available in the data set.

**Table 3:** An overview of numerical results related to analysis in Section 4.3 (PILATUS) Total number of frames 453 231.

Procedure	No. of hits	Hit fraction (%)	No. of indexed frames	Indexing rate (%)
RPF with data set mask	452 259	99.78	29 777	6.58
RPF with RMM	282 452	62.31	34 550	12.23
PF8 with data set mask†	453 231	100.0	23 864	5.26
PF8 with RMM	90 479	19.96	27 425	30.31

† Reproduced using the output available in the data set.

## Appendix A Robust Gaussian fitting

In this appendix we review the underlying mathematical operations used in RMM.

Given *X* = {*x*(*j*)}, *j* = 1,…, *N*, where  , a set of *m*-dimensional data points, and using a model (with ρ parameters) parametrized by  , the task is to segment a group of data points according to their relationship to each other.

The group of strongly correlating data points (according to the fitted model) are usually called the data structure. The size of this data structure, *i.e.* the number of data points in the group, is initially unknown. However, an initial guess for structure size can help in solving the problem and we denote this by *k*. The least *k*th order statistics (LkOS) introduced by Bab-Hadiashar & Hoseinnezhad (2008 ▸) is the chosen cost function for fitting the model to the data, and an optimization algorithm is incorporated that aims to minimize  where *d*_[*k*],θ_ is the *k*th sorted residual with respect to the model with parameters θ. Given a sampled subset of *X* with size ρ + 4 [that we choose to adopt from the work of Tennakoon *et al.* (2016 ▸)] called *e*, the parameters of the model are obtained by fitting it non-robustly to *x*_*e*_ where θ_*e*_ =  . To make this non-robust fit to the small subset, first a distance measure  =  is defined for all *x* in *e* and is used to calculate the residuals (fitting errors) for all *N* data points. Note that *N* is the number of data points given to the robust estimator function as used in Section 3.1. A cost function  is then defined using some norm over the residuals, *i.e.*  =  (where  is a non-robust cost function). The noise of the data points is assumed to be independent and each follows the same normal distribution  with variance σ. The parameter norm is usually 2, which turns the optimization into a linear regression problem, the solution of which can be obtained in closed form enabling fast computation. The evaluation function  is defined depending on the number of parameters of the geometric model. For example (in the calculation of Feature 2 in the main text), for three-dimensional data points (*m* = 3, where the locations of the pixels and their intensities matter) the model can be a four-dimensional plane or a more complex surface which will be affected by how  is formulated (shown in Fig. 10 ▸).

The positive integer *k* is the initial guess of the number of inliers out of *N* total data points and is initialized to a value more than 0.5*N* in all the applications in this paper. This means that we initially assume that half of the pixels are good pixels in any given reasonably large window on the detector pixels. It is notable that, because of this assumption, the estimates of the model parameters are independent of σ and therefore of λ. The LkOS cost function, as described by Rousseeuw & Leroy (2003 ▸) and Chin *et al.* (2009 ▸), has its minima where the underlying structure is highly likely to be in the parameter space. In other words, there is a probability density function (not in closed form) that models how likely it is for input data points to take different values, and the samples leading to models with low LkOS cost are generated according to this density function. As such, samples are made relative to how well the model fitted to them explains the data with high probability, leading to a good fit to the data. This allows the detection of structures in the presence of outliers, especially since this cost function is biased towards structures with low-cost fitting errors, with little attention to their size. However, the local minimization task is challenging, since the cost function is highly nonlinear due to the sorting step, making it unfeasible to obtain its derivatives analytically. Bab-Hadiashar & Hoseinnezhad (2008 ▸) introduced the fast least *k*th order statistics (FLkOS) algorithm, which uses approximate second derivatives (similar to Newton’s method) to find a local minimum rapidly. This is the robust optimization method that we use to find the minimum of the LkOS cost function  . The robust optimization method begins with an initial subset *e* of size  which is randomly selected from the input data and non-robustly fits a geometric model to it. For the robust optimizer, the initial sample does not have to be purely made from the target structure (the target structure would be the subset of normally behaving data points among all data points given to the robust estimator function). Tennakoon *et al.* (2016 ▸) showed that this robust method can easily be extended to use larger sample sizes and still minimize the cost function effectively. This is particularly important when it comes to sampling from the challenging underlying probability density function assumed to be modelling the data. Such a density function would be a convolution of many kinds of noise functions, including a Poisson density (due to counting photons) convolved with the noise density of the detector that changes with temperature. It was shown by Sadri *et al.* (2018 ▸) that the optimization method is capable of finding the mode of a skewed density function (suitable for dealing with Poisson processes). After the mode is detected, an i.i.d (independent and identically distributed) Gaussian noise is assumed for all data points in *X* to find inliers, and the non-robust average and standard deviation of the inliers (with noise levels less than a given threshold) is given as the output of the robust estimator function.

The FLkOS algorithm uses the sorting indices of the entire input data *x* to perform the robust optimization iteratively as follows. The squared fitting errors  are calculated for each data point in *x* (according to model θ obtained by non-robust fitting of the geometric model to an initial random sample). The set of indices *I* is found to sort the set of *d*_*i*_ (*I*_*i*_ ≤ *I*_*j*_ if *d*_*i*_ ≤ *d*_*j*_). Since outliers are present, the optimization of the LkOS cost function, intuitively, is such that it minimizes the residual of the furthest inlier (the inlier with the largest fitting error). The strategy of FLkOS embeds the calculation of derivatives of the cost function and applies the gradient descent by sampling from sorted residuals and letting the new subset *e* be *e* =  . This then runs iteratively. By iterating only once, the percentile of input values would be found (such as the median for *k* = *N*/2). However, we propose to use 12 steps of the robust optimization according to the work of Bab-Hadiashar & Hoseinnezhad (2008 ▸). Given the final optimized model parameters, *d*_*i*_ is calculated with respect to the model. A χ square is fitted to the residuals from which the σ of the Gaussian structure can be calculated. This is done by finding the first sorted residual *d*_[*k*]_ such that  . These *k* data points (the final estimated structure size) with the lowest fitting errors are inliers of the model whose non-robust average and standard deviation are provided as the output of the above procedure, *i.e.*  =  and  =  . Finally, the statistical separability of the *i*th data point from data points in *X* is SNR(*i*) =  .

To clarify the above, an example is presented in Fig. 11 ▸. In the left-hand panel a set *X* of randomly generated data points are shown. The values of *N* = 150 data points are shown, where *N*_in_ = 100 of them are identically independently distributed by a normal probability density with average μ_in_ = 20 and standard deviation σ_in_ = 5. *N*_out_ = 50 data points are distributed according to a uniform density with a lower bound of −20 and an upper bound of 180. The non-robust average and upper thresholds are defined by μ and μ + 3σ, respectively. The robust average, upper and lower thresholds are defined by μ_in_, μ_in_ + 3σ_in_ and μ_in_ − 3σ_in_, respectively. The settings ρ = 8, *k* = *N*/2 and λ = 3 were applied. This method estimates the thresholds more accurately than the non-robust way.

## Appendix B Discussion of λ

In this appendix we discuss how changing the parameter λ affects the results of the analysis pipeline described in Section 4.1, using the RPF software (Hadian-Jazi *et al.*, 2021 ▸).

Increasing λ allows the scale of the noise to be biased by the outliers which, in the task of making the bad-pixel mask, might lead to including bad pixels in the normal group. A part of the data set (from run 96, sequences 2 and 5, comprising 180 000 patterns from which around 44 000 were indexed) was selected for this test. The effect of the mask is shown using self-consistency statistics for two specific resolution values. As can be seen from Fig. 12 ▸, the statistics are insensitive to the value of λ over the range 7 ≤ λ ≤ 12. Based on this figure, we recommend a value of λ = 8.0. As λ gets smaller, good pixels are labelled as bad, and as it gets larger, bad pixels are labelled good.
